# *Legionella* Detection in Environmental Water Samples Submitted to the Gyeonggi-do Institute of Health and Environment, 2023–2025: Sample-type Differences and the Increasing Contribution of Cooling-tower Water to Positive Samples

**DOI:** 10.56786/PHWR.2026.19.39.2

**Published:** 2026-10-15

**Authors:** Shin-Hye Kwak, Hye-Jung Kwon, Sang-Tae Kim, Hye-Rim Song, Ye-Jin Hong, I-Hyeon Kim, Jin-A Jeong, Myung-Gil Kim

**Affiliations:** Division of Infectious Disease Research, Gyeonggi-do Institute of Health and Environment Suwon Korea

**Keywords:** Legionellosis, *Legionella*, Environmental monitoring, Water supply, Water microbiology

## Abstract

**Objectives**: *Legionella* spp. proliferate in artificial water systems, such as cooling towers and water supply and hot water systems of buildings, causing infection through the inhalation of contaminated aerosols. We analyzed the detection characteristics of *Legionella* spp. within environmental water samples submitted to the Gyeonggi-do Institute of Health and Environment, the Republic of Korea, to determine management priorities.

**Methods**: We analyzed 9,172 *Legionella* spp. testing records for environmental water samples received from January 2023 through December 2025, summarized by sample type, purpose, submission route, species, and concentration. Chi-square and Cochran–Armitage trend tests were used.

**Results**: *Legionella* spp. were detected in 570/9,172 samples (6.21%; 95% confidence interval, 5.74–6.73%). Detection differed significantly by sample type (p<0.001)—bathtub water: 18.30%, hot water: 11.03%, cooling-tower water: 5.53%, other water: 3.53%, and cold water: 1.28%; the ranking excluding other water was consistent across 3 years. Cooling-tower water accounted for 47.2% of positives, increasing significantly from 34.2% in 2023 to 59.6% in 2025 (p for trend<0.001); after standardization for sample-type composition, the increase was smaller (43.2% to 53.1%). Requester-collected submissions comprised 96.4% (4,691/4,865) of cooling-tower samples; detection rates were 14.94% (26/174) for officially-collected and 5.18% (243/4,691) for requester-collected samples. Cooling-tower water accounted for 55.6% of positives >10,000 CFU/L.

**Conclusions**: Bathtub and hot water systems require preventive management given their high detection proportions, whereas cooling towers require surveillance priority given their large and increasing contribution to positives. Priorities should combine sample-type detection rates with the size and trend of positive counts. As cooling-tower testing relies largely on voluntary requests, risk-targeted official testing should complement it.

**Key messages**

**① What is known previously?**

*Legionella* spp. can proliferate in artificial water systems, including cooling towers and building water systems; temperature, disinfection, and cleaning are central to prevention.

**② What new information is presented?**

Detection ranked consistently as bathtub water, hot water, cooling-tower water, and cold water. Cooling towers accounted for 47.2% of positives, increasing from 34.2% in 2023 to 59.6% in 2025 (43.2% to 53.1% after standardization for sample-type composition); 96.4% of cooling-tower samples were voluntary submissions.

**③ What are implications?**

Priorities should combine detection probability and positive burden: preventive management for bathtub/hot water systems and surveillance priority for cooling towers, complemented by risk-targeted official testing.

## Introduction

Legionellosis is an acute respiratory disease caused by *Legionella* species infection, and is a Group 3 notifiable infectious disease that must be reported within 24 hours in accordance with the Infectious Disease Control and Prevention Act [1]. *Legionella* exists in natural environments and artificial water systems, and human infection mainly occurs through the inhalation of aerosols generated from contaminated water [2-4]. Clinically, it manifests as a pneumonic form accompanied by fever, cough, and dyspnea, and a mild Pontiac fever that usually resolves spontaneously, with a high risk of progressing to severe disease in the elderly, individuals with chronic diseases, and immunocompromised individuals [2].

*Legionella* can proliferate in warm, stagnant water and biofilm-formed environments. Thus, cooling-tower water, bath water in public bath facilities, and building water supply facilities and hot water supply facilities are the main targets of management [3,4]. In the Republic of Korea, reported cases have increased since 2016, and 560 cases (provisional) were reported in 2025 [5,6]. The Korea Disease Control and Prevention Agency (KDCA) presents environmental management for each facility and post-testing measures through environmental inspection plans for multi-use facilities and the 2026 Guideline for the Management of Legionellosis [6]. The Gyeonggi-do Institute of Health and Environment receives both official samples collected by public officials in charge and client-requested samples directly collected by requestors, such as facility managers and users. Since the two pathways differ in terms of the sampling entity and target selection, it is necessary to interpret them as data describing the testing composition and management demand rather than as a simple comparison of detection rates.

This report aimed to analyze the results of *Legionella* culture tests on environmental water samples received from 2023 to 2025 to determine the detection rate by sample type, yearly changes in the proportion of cooling-tower water among positive samples, and the composition by request pathway, and to suggest priorities for environmental water system management considering the likelihood of detection and the burden of positive results.

## Methods

### 1. Target Samples and Data Sources

This study analyzed the testing and inspection records of *Legionella* environmental water samples received by the Gyeonggi-do Institute of Health and Environment from January 1, 2023 to December 31, 2025. The year was assigned based on the receipt date. Official samples collected by public officials with jurisdiction were classified into samples collected for planned preventive inspections and epidemiological investigation samples collected following the occurrence of cases. Client-requested samples were voluntarily submitted samples directly collected and submitted to the institute by requesters, such as facility managers and users. The unit of analysis was the sample from each water collection point rather than the facility, and the detection rate referred to the proportion of positive results among the received samples.

### 2. Test Method

Pretreatment, concentration, and culture of samples were performed in accordance with the KDCA “2026 Guideline for the Management of Legionellosis” and the National Institute of Environmental Research (NIER) “Analysis method for *Legio­nella* in environment” applied at the time of testing [6,7]. After culturing on glycine, vancomycin, polymyxin B, cycloheximide (GVPC) selective agar, buffered charcoal yeast extract (BCYE) agar, and BCYE agar without L-cysteine (BCYE-Cys), suspected colonies were identified, and colony-forming units (CFUs) were calculated and presented in terms of CFU/L. The limit of detection of the culture test was 200 CFU/L. Species identification (16S ribosomal ribonucleic acid [16S rRNA] and macrophage infectivity potentiator [*mip*] gene polymerase chain reaction and sequencing) of suspected colonies confirmed in culture was not uniformly applied to all positive colonies, but was selectively performed only for recirculating filtered bath water samples and samples deemed by epidemiological investigators to require identification.

### 3. Definition of Detection and Variables

Detection (positive) was defined when *Legionella* colonies were confirmed on selective media culture, and for water system samples where quantification was possible, a value of 200 CFU/L or more was considered positive detection. Based on the details of the test log, sample types were classified into cooling-tower water, hot water, bathtub water, cold water, and other. Surface swab samples from water taps and showerheads were included in other. Because swab samples do not have a collected water volume, quantitative conversion is not possible, so they were processed as qualitative results, and the corresponding 12 positives were excluded from the concentration analysis. For 558 positive samples with confirmed quantitative values, concentrations were categorized into <1,000, 1,000–10,000, >10,000–<100,000, and ≥100,000 CFU/L. The interval boundaries were determined in accordance with the action thresholds of the guidelines. The 2026 guidelines present different phased measures for cooling-tower water and cold and hot water in water supply facilities, and since a water quality standard of 1,000 CFU/L is applied to recirculating bath water in public baths, the concentration intervals were interpreted according to sample types [6].

### 4. Statistical Analysis

The detection rate was calculated by dividing the number of detected samples by the number of tested samples. Differences between sample types were evaluated using the Pearson chi-square test, and Yates’ continuity correction was applied to 2×2 tables for comparisons of sample sources and for comparisons of cooling-tower water sample sources. The detection rate ratio by type was calculated using cold water as the reference. The yearly trend in the proportion of cooling-tower water among all positives was evaluated using the Cochran–Armitage trend test, and the Wilson 95% confidence interval (CI) was presented for key detection rates. Testing by request pathway was performed to compare the distribution of detection rates between the two receipt groups, and since target selection, the sampling entity, and the retesting method differed, it was not interpreted as an evaluation of the effect of the pathway itself or the superiority of the sampling method. Since the proportion of cooling-tower water among all positives is influenced by the annual sample type composition, direct standardization using the combined 3-year sample type composition as the standard was performed as a sensitivity analysis and presented alongside the observed values. The directly standardized proportion is calculated by applying the detection rate by sample type of each year to the 3-year combined sample type composition, representing the expected proportion of cooling-tower water assuming that the sample composition was identical every year. Through this approach, we aimed to separate the observed change in proportion into the portion attributable to changes in sample type composition and the portion attributable to changes in type-specific detection rates. The 95% CIs of the directly standardized proportions were calculated using a bootstrap based on binomial resampling for each sample type and year stratum. Analysis was performed using Python 3.12 (Python Software Foundation), and a two-sided p-value of <0.05 was considered statistically significant.

## Results

### 1. Overall and Annual Detection Status

Among the 9,172 analyzed samples (3,643 official samples and 5,529 client-requested samples), *Legionella* was detected in 570 samples (6.21%, 95% CI 5.74–6.73%). The annual detection rates were 6.98% (193 positive/2,764 samples) in 2023, 4.22% (137 positive/3,246 samples) in 2024, and 7.59% (240 positive/3,162 samples) in 2025, showing no apparent trend. The 3-year combined detection rates for official samples and client-requested samples were 6.73% and 5.88%, respectively, with no significant difference (Table 1). By purpose of testing, official epidemiological investigation samples showed the highest detection rate at 9.82% (120 positive/1,222 samples), followed by client-requested samples at 5.88% (325 positive/5,529 samples) and official preventive samples at 5.16% (125 positive/2,421 samples). The annual values were unadjusted proportions reflecting changes in the composition of sample types and request pathways and were thus presented descriptively.

### 2. Detection Status by Sample Type

The detection rate by sample type showed significant differences among types, with bathtub water at 18.30% (86 positive/470 samples, 95% CI 15.06–22.05%), hot water at 11.03% (166 positive/1,505 samples, 95% CI 9.54–12.72%), cooling-tower water at 5.53% (269 positive/4,865 samples, 95% CI 4.92–6.21%), other at 3.53% (30 positive/851 samples), and cold water at 1.28% (19 positive/1,481 samples) (p<0.001). Compared to cold water, the detection rate ratio was 14.3-fold for bathtub water, 8.6-fold for hot water, and 4.3-fold for cooling-tower water. The order of bathtub water, hot water, cooling-tower water, and cold water remained the same for the 3 years, but the ranking of other varied by year (Table 2, Figure 1). The “Other” category is a mixed category that includes water systems with different characteristics, such as fountain water, storage-tank water, and filter water, along with surface swab samples for which quantitation is not possible. Cooling-tower water accounted for 53.0% of all tests and 47.2% of all positives. The proportion of cooling-tower water among all positives significantly increased from 34.2% in 2023 to 43.8% in 2024 and 59.6% in 2025 (trend test p<0.001) (Figure 2). However, the proportion of cooling-tower water out of the total tests also increased to 42.8% in 2023, 56.7% in 2024, and 58.3% in 2025, and the number of tests for hot water and cold water decreased. The proportion of cooling-tower water, directly standardized to the 3-year combined sample type composition, was 43.2% in 2023, 41.5% in 2024, and 53.1% in 2025; although it did not increase consistently, it was the highest in 2025. Approximately two-thirds of the observed increase was attributed to changes in sample composition, and approximately one-third was attributed to changes in type-specific detection rates. The annual trend of the detection rate in cooling-tower water samples was significant (5.58% in 2023, 3.26% in 2024, and 7.75% in 2025; trend test p=0.001).

### 3. Cooling-tower Water Testing Composition

Of the 4,865 cooling-tower water samples, 4,691 (96.4%) were client-requested samples and 174 (3.6%) were official samples. Of the 269 positive cooling-tower water samples, 243 (90.3%) were client-requested samples. The detection rate of official cooling-tower water was 14.94% (26 positives/174 samples, 95% CI 10.41–20.99%), and that of client-requested samples was 5.18% (243 positives/4,691 samples, 95% CI 4.58–5.85%), and the difference in the rates was significant (Figure 3).

### 4. Bacterial Species and Quantitative Concentration

Among the 570 positives, in 131 samples for which the species was identified by sequencing, *Legionella pneumophila*, *Legionella qingyii*, *Legionella spiritensis*, *Legionella anisa*, and *L. pneumophila* subsp. *pascullei* were identified in 112 (85.5%), 6 (4.6%), 4 (3.1%), 3 (2.3%), and 3 (2.3%) samples, respectively. The *L. pneumophila* group, including *L. pneumophila* subsp. *pascullei*, accounted for 115 samples (87.8%). The median of 558 positive samples (excluding 12 surface swab samples) for which quantitative conversion was possible was 1,800 CFU/L (range 200–1,024,000 CFU/L). The concentrations of 350 samples (62.7%) were 1,000 CFU/L or higher. Among the 133 samples with concentrations exceeding 10,000 CFU/L, cooling-tower water accounted for 74 samples (55.6%) (Table 3).

## Discussion

Although bathtub water and hot water had the highest detection rates, cooling-tower water accounted for 53.0% of all tests and 47.2% of positives, and its proportion among all positives also increased from 34.2% in 2023 to 59.6% in 2025 (from 43.2% to 53.1% when standardizing sample composition). The core point presented by this study is that the sample-level detection rate and overall burden of positive results represent distinct management indicators. Therefore, rather than prioritizing environmental water system management solely based on a single detection rate ranking, the sample-level likelihood of detection and the magnitude and trend of the number of positive samples must be evaluated together.

The ranking of detection rates for bathtub water, hot water, cooling-tower water, and cold water was consistent over the 3 years. This finding was consistent with the results of previous studies showing that warm, stagnant water systems and biofilms are suitable for the proliferation of *Legionella* [3,4]. Since bathtub water and hot water have a high likelihood of detection even when the testing volume is relatively low, they are priority targets for preventive water system management focusing on water temperature, disinfection, circulation, and cleaning. However, our findings reflected the detection rate of received samples and do not directly indicate the facility-level contamination rate or infection risk. Since “Other” is a mixed category comprising water systems with different characteristics and non-quantifiable surface swab samples, annual fluctuations in rankings were not interpreted as the detection characteristics of a single water system.

Although the detection rate for cooling-tower water was 5.53%, it accounted for a large proportion of positive samples and high-concentration positives, and the proportion of cooling-tower water among all positives significantly increased over the 3 years. This finding indicates that cooling-tower water poses a surveillance burden that is not revealed by the detection rate ranking alone. In addition, since 96.4% of cooling-tower water tests and 90.3% of positives were voluntary client-requested samples, the currently identified burden of positive results for cooling-tower water heavily depends on client demand. The observation of a high detection rate in official cooling-tower water suggests the possibility that high-risk facilities were included in the target selection by public officials. However, as the number of tests for official cooling-tower water was small at 174, resulting in a wide 95% CI of 10.41–20.99% for the detection rate, it is difficult to quantitatively confirm this difference, along with the differences in target selection and sampling entities between the two routes. For official planned inspections, rather than a uniform expansion of the number of tests, consideration may be given to targeting high-risk facilities that are not captured through voluntary client requests, based on risk factors such as facility types, the likelihood of aerosol exposure, and past detection history.

Of the 558 positive samples for which quantitative conversion was possible, the concentrations of 350 samples (62.7%) were 1,000 CFU/L or higher, and cooling-tower water accounted for 74 (55.6%) of the 133 samples with concentrations exceeding 10,000 CFU/L. This finding shows that the importance of cooling-tower water is evident not only in the testing volume but also in the high-concentration burden of positive results. However, because different action thresholds apply to cooling-tower water, cold and hot water in water supply facilities, and bath water in public baths [6], the concentration must be interpreted according to the standards for each water system type. Among the 131 samples with recorded identification results, the *L. pneumophila* group was detected in 87.8% of samples. However, since species identification was selectively performed only on a portion of samples, such as recirculating filtered bath water and epidemiological investigation samples, the identified species distribution does not represent all positive samples.

This analysis has four limitations. First, because official samples and voluntary client-requested samples received by a single institution were analyzed, the contamination rate of all facilities in Gyeonggi-do cannot be estimated. Second, official samples and client-requested samples differ in target selection, the sampling entity, and retesting methods, so differences in detection rates by request pathway cannot be causally interpreted. Third, because facility management variables, such as water temperature, residual chlorine, piping conditions, and cleaning history, could not be analyzed, the specific causes of the differences in detection could not be evaluated. Fourth, the proportion of cooling-tower water among all positives was affected by the annual sample composition. Therefore, a significant portion of the observed increase is explained by changes in composition (the increase in 2023–2024 disappeared after standardization, but the 2025 proportion was the highest even after standardization). Therefore, this proportion reflects the composition of the burden of positive results processed by the institute and does not directly imply an increased risk of contamination in cooling-tower facilities.

In conclusion, this study showed that “where the detection rate is high” and “where the burden of positive results accumulates substantially” are not the same. Bathtub water and hot water are priority targets for preventive water system management, and cooling-tower water accounts for the largest proportion of overall positives; as its proportion is also increasing, it is a priority target for surveillance. Therefore, environmental water system management should be designed with dual priorities that simultaneously apply the detection rate by sample type and the magnitude and trend of the number of positive samples, and official planned inspections need to strengthen their role in supplementing high-risk cooling towers that are not captured through voluntary client requests. When such environmental surveillance leads to the early identification of and preemptive measures for high-risk environmental water systems, it can contribute to reducing the occurrence of legionellosis cases.

## Figures and Tables

**Figure 1:**
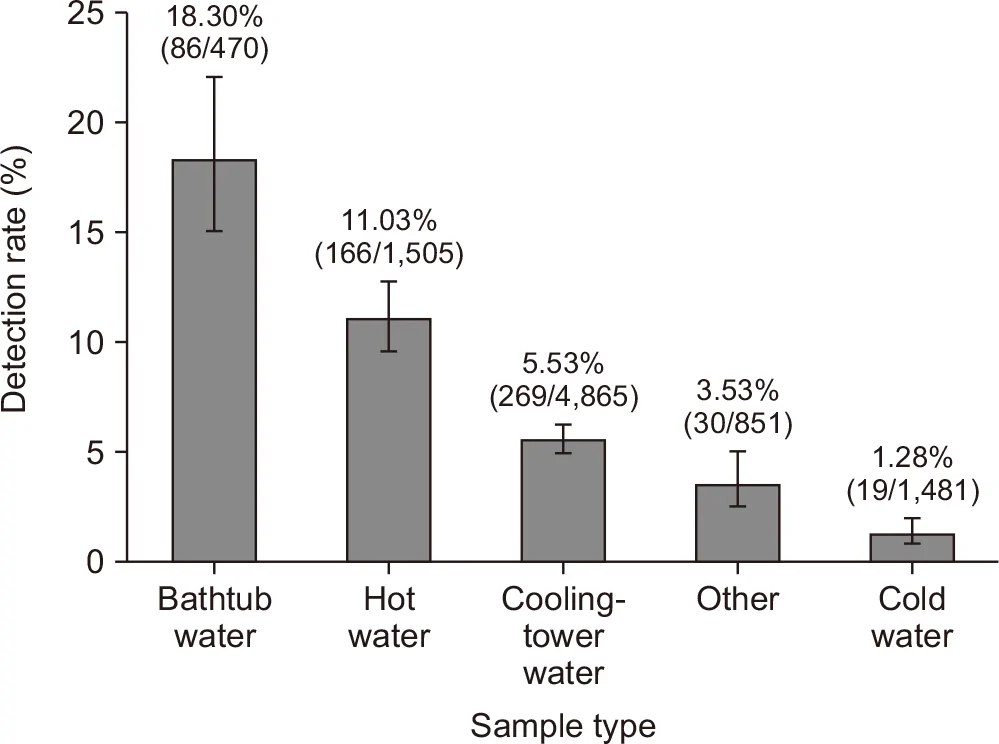
*Legionella* spp. detection rate by sample type in environmental water samples submitted to the Gyeonggi-do Institute of Health and Environment, 2023–2025 combined Error bars indicate Wilson 95% confidence intervals.

**Figure 2:**
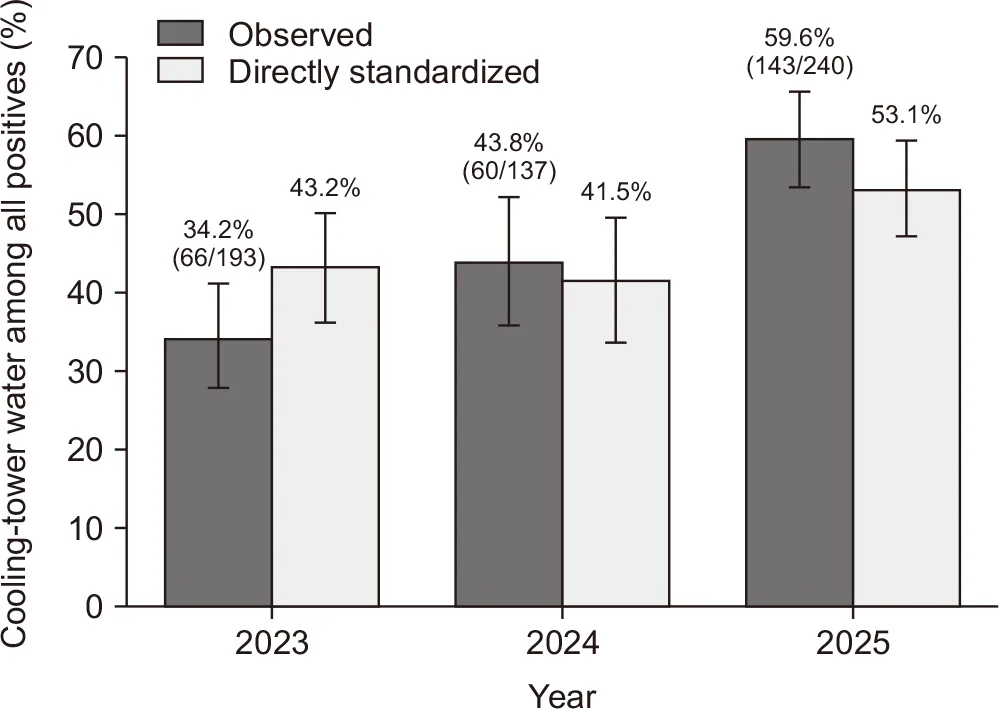
Observed and directly standardized proportions of cooling-tower water among *Legionella* spp.-positive samples by year, Gyeonggi-do Institute of Health and Environment, 2023–2025 Dark bars indicate observed proportions—labels show the proportion and cooling-tower positives/total positives; light-colored bars indicate proportions directly standardized to the 3-year pooled sample-type composition. Error bars represent 95% confidence intervals, calculated by using the Wilson method for observed values and by a binomial-resampling bootstrap for standardized values.

**Figure 3:**
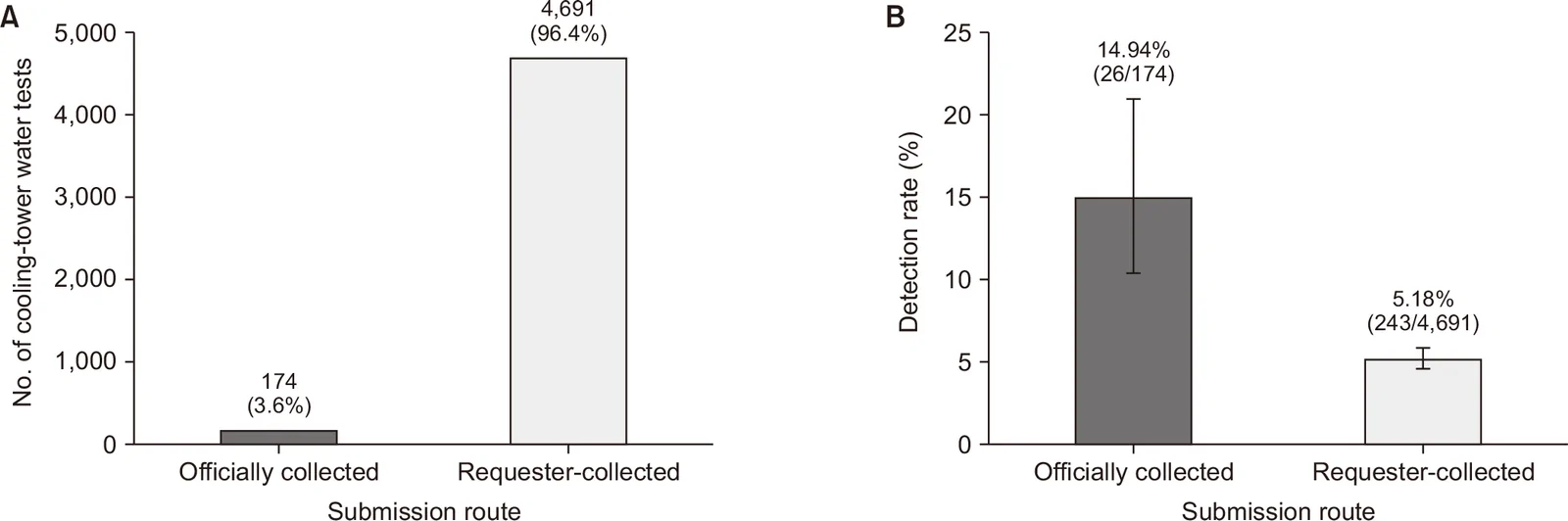
Cooling-tower water testing and detection by submission route, Gyeonggi-do Institute of Health and Environment, 2023–2025 (A) No. of cooling-tower water tests, (B) cooling-tower water detection rate. Officially-collected samples included 173 preventive surveillance samples and 1 epidemiological investigation sample; error bars indicate Wilson 95% confidence intervals.

**Table 1:** Annual *Legionella* testing and detection by submission route, Gyeonggi-do Institute of Health and Environment, 2023–2025

Submission route	Yeara)	No. of samples tested	No. of positive samples	Detection rate (%)b)
Officially-collected	2023	1,441	109	7.56
	2024	1,243	54	4.34
	2025	959	82	8.55
Requester-collected	2023	1,323	84	6.35
	2024	2,003	83	4.14
	2025	2,203	158	7.17
Total	2023	2,764	193	6.98
	2024	3,246	137	4.22
	2025	3,162	240	7.59
	2023–2025	9,172	570	6.21

^a)^Year was assigned by the date of receipt; one sample with a missing receipt date was assigned by its accession number. ^b)^Detection rate=number of positive samples/number of tested samples×100.

**Table 2:** *Legionella* detection by sample type and year, Gyeonggi-do Institute of Health and Environment, 2023–2025

Sample type	2023a)	2024	2025	Total tested	Detection rate (%)
Bathtub water	27/138 (19.57)	34/180 (18.89)	25/152 (16.45)	470	18.30
Hot water	79/588 (13.44)	32/501 (6.39)	55/416 (13.22)	1,505	11.03
Cooling-tower water	66/1,182 (5.58)	60/1,839 (3.26)	143/1,844 (7.75)	4,865	5.53
Cold water	5/583 (0.86)	2/496 (0.40)	12/402 (2.99)	1,481	1.28
Otherb)	16/273 (5.86)	9/230 (3.91)	5/348 (1.44)	851	3.53

^a)^Each yearly cell shows the number of positive samples/number of tested samples (detection rate). ^b)^Fountain water, storage tank water, and filter water as well as surface swab samples from faucets and showerheads.

**Table 3:** Identified *Legionella* species and concentration distribution among positive samples, Gyeonggi-do Institute of Health and Environment, 2023–2025

Category	n (%)
Identified species (n=131)a)	
*Legionella pneumophila*	112 (85.5)
*Legionella qingyii*	6 (4.6)
*Legionella spiritensis*	4 (3.1)
*Legionella anisa*	3 (2.3)
*Legionella pneumophila* subsp. *pascullei*	3 (2.3)
Othersb)	3 (2.3)
Concentration category (n=558) (CFU/L)c)	
<1,000	208 (37.3)
1,000–10,000d)	217 (38.9)
>10,000–<100,000	117 (21.0)
≥100,000	16 (2.9)

Concentration categories are descriptive and must be interpreted separately from thresholds applied to each water-system type. Values are the number of samples (%). Percentages may not sum to 100% due to rounding. CFU=colony-forming unit. ^a)^Based on the 131 positive samples with sequencing results available. ^b)^Others: *Legionella bozemanii*, *Legionella erythra*, and *Legionella micdadei* (1 each). ^c)^Based on 558 positive samples, excluding 12 swab samples for which quantification was not possible. ^d)^Concentration categories are mutually exclusive; the 1,000–10,000 CFU/L category includes 10,000 CFU/L.
