# Supplementary material for: Legionella Detection in Environmental Water Samples Submitted to the Gyeonggi-do Institute of Health and Environment, : 2023–2025: Sample-type Differences and the Increasing Contribution of Cooling-tower Water to Positive Samples
Source: Public Health Wkly Rep. 2026 Oct 15;19(39):1667–83. doi: 10.56786/PHWR.2026.19.39.2 (PMC13672474; doi:10.56786/PHWR.2026.19.39.2)
Supplement: Translation

# Translation: 2023–2025년 경기도보건환경연구원 접수 환경수계 검체의 레지오넬라균 검출 특성: 검체 유형별 차이와 냉각탑수 양성 비중의 증가

**Authors:** 신혜 곽, 혜정 권, 상태 김, 혜림 송, 예진 홍, 이현 김, 진아 정, 명길 김

**Affiliations:** 경기도보건환경연구원 감염병연구부

**Keywords:** 레지오넬라증, 레지오넬라균, 환경감시, 급수, 수질 미생물학

## Abstract

**목적:**

레지오넬라균은 냉각탑과 건물 급수 및 급탕시설 등 인공 수계에서 증식하며, 오염된 물에서 발생한 에어로졸의 흡입으로 감염을 일으킨다. 경기도보건환경연구원에 접수된 환경수계 검체의 레지오넬라균 검출 특성을 분석하여 환경수계 관리의 우선순위를 제시하고자 하였다.

**방법:**

2023년 1월 1일부터 2025년 12월 31일까지 접수된 환경수계 검체 9,172건의 레지오넬라균 시험검사 기록을 분석하였다. 공무원이 채취한 관원 검체와 의뢰자가 직접 채취한 민원 검체를 구분하고 검체 유형과 검사목적, 균종, 정량농도별로 요약하였다. 검출률 차이는 카이제곱검정으로, 전체 양성 중 냉각탑수 비중의 연도 추이는 Cochran–Armitage 경향검정으로 평가하였다.

**결과:**

전체 9,172건 중 570건(6.21%, 95% 신뢰구간 5.74–6.73%)에서 레지오넬라균이 검출되었다. 검체 유형별 검출률은 욕조수 18.30%, 온수 11.03%, 냉각탑수 5.53%, 기타 3.53%, 냉수 1.28%로 유형 간 유의한 차이가 있었고(p<0.001), 기타를 제외한 검출률 순위(욕조수, 온수, 냉각탑수, 냉수)는 3년간 일관되게 유지되었다. 냉각탑수는 전체 양성의 47.2% (269/570건)를 차지했으며, 그 비중은 2023년 34.2%에서 2024년 43.8%, 2025년 59.6%로 유의하게 증가하였다(경향검정 p<0.001). 다만 이는 검체 구성을 보정하지 않은 값으로, 3년 합산 구성으로 직접표준화한 후 43.2%에서 53.1%로 증가폭이 축소되었다. 냉각탑수 검체의 96.4% (4,691/4,865건)는 민원 검체였고, 관원과 민원 냉각탑수의 검출률은 각각 14.94% (26/174건)와 5.18% (243/4,691건)였다. 10,000 CFU/L 초과 양성 133건 중 냉각탑수는 74건(55.6%)이었다.

**결론:**

욕조수와 온수는 검체당 레지오넬라균 검출 가능성이 높아 예방적 수계관리의 우선 대상이며, 냉각탑수는 전체 양성의 가장 큰 비중을 차지하고 그 비중도 증가하여 감시의 우선 대상이다. 따라서 검체 유형별 검출률과 양성 건수의 규모와 추이를 함께 적용하는 이중 우선순위가 필요하다. 냉각탑수 검사의 대부분이 자율의뢰에 의존하므로 관원 계획검사는 민원 수요로 포착되지 않는 고위험 시설을 표적 선정하여 보완할 필요가 있다.

**핵심요약**

**① 이전에 알려진 내용은?**

레지오넬라균은 냉각탑과 건물 급수시설과 급탕시설 등 인공 수계에서 증식할 수 있어 수온과 소독, 청소 관리가 중요하다.

**② 새로이 알게 된 내용은?**

2023–2025년 9,172건의 검출률은 6.21%였고, 욕조수, 온수, 냉각탑수, 냉수의 순서는 3년간 일관되었다. 냉각탑수는 전체 양성의 47.2%를 차지했으며 그 비중은 34.2%에서 59.6%로 유의하게 증가하였다(p<0.001). 연도별 검체 유형 구성을 직접표준화한 후 냉각탑수 비중은 43.2%에서 53.1%로 증가하였다. 냉각탑수 검사의 96.4%는 자율의뢰 민원 검체였다.

**③ 시사점은?**

관리 우선순위는 검출률과 양성 부담을 함께 고려해야 한다. 욕조수와 온수는 예방적 수계관리의 우선 대상으로, 냉각탑수는 감시의 우선 대상으로 관리하고, 관원 계획검사는 민원 자율의뢰로 포착되지 않는 고위험 시설을 보완해야 한다.

## 서 론

레지오넬라증은 레지오넬라균(*Legionella* species) 감염에 의한 급성 호흡기 질환으로, 「감염병의 예방 및 관리에 관한 법률」에 따라 24시간 이내 신고하여야 하는 제3급 법정감염병이다[1]. 레지오넬라균은 자연환경과 인공 수계에 존재하며, 인체 감염은 주로 오염된 물에서 생성된 에어로졸의 흡입으로 발생한다[2-4]. 임상적으로는 발열과 기침, 호흡곤란을 동반하는 폐렴형과 대개 자연 회복되는 경증의 폰티악열로 나타나며, 고령자와 만성질환자, 면역저하자에서 중증으로 진행할 위험이 높다[2].

레지오넬라균은 따뜻하고 정체된 물과 생물막이 형성된 환경에서 증식할 수 있어 냉각탑수, 목욕장 욕조수, 건물 급수시설과 급탕시설이 주요 관리 대상이다[3,4]. 국내 신고는 2016년 이후 증가하였고 2025년에는 560명(잠정)이 신고되어 높은 수준을 보였다[5,6]. 질병관리청은 다중이용시설 환경검사 계획과 「레지오넬라증 관리지침」을 통해 시설별 환경관리와 검사 후 조치를 제시하고 있다[6]. 경기도보건환경연구원에는 관할 공무원이 채취한 관원 검체와 시설 관리자와 이용자 등 의뢰자가 직접 채취한 민원 검체가 함께 접수된다. 두 경로는 채취 주체와 대상 선정이 다르므로, 검출률의 단순 비교보다는 검사 구성과 관리 수요를 기술하는 자료로 해석할 필요가 있다.

이 보고는 2023–2025년 접수된 환경수계 검체의 레지오넬라균 배양검사 결과를 분석하여 검체 유형별 검출률과 냉각탑수 양성 비중의 연도별 변화 및 의뢰 경로별 구성을 파악하고, 검출 가능성과 양성 부담을 함께 고려한 환경수계 관리의 우선순위를 제시하고자 하였다.

## 방 법

### 1. 대상 검체 및 자료원

이 연구는 2023년 1월 1일부터 2025년 12월 31일까지 경기도보건환경연구원에 접수된 환경수계 검체의 레지오넬라균 시험검사 기록을 분석하였다. 연도는 접수일을 기준으로 배정하였다. 관원 검체는 관할 공무원이 채취한 예방 목적의 계획검사와 환자 발생에 따른 역학조사 검체로 구분하였다. 민원 검체는 시설 관리자와 이용자 등 의뢰자가 직접 채취하여 연구원에 제출한 자율의뢰 검체이다. 분석 단위는 시설이 아닌 개별 채수지점의 검체이며, 검출률은 접수된 검체 중 양성 비율을 의미한다.

### 2. 검사 방법

검체의 전처리와 농축, 배양은 검사 당시 적용한 질병관리청 「레지오넬라증 관리지침」 및 국립환경과학원 「환경 중 레지오넬라 표준분석법」에 따라 수행하였다[6,7]. 선택배지인 GVPC (glycine, vancomycin, polymyxin B, cycloheximide) 배지, BCYE (buffered charcoal yeast extract) 배지, L-cysteine을 제외한 BCYE-Cys 배지에서 배양한 후 의심 집락을 확인하고 집락형성단위(colony-forming unit, CFU)를 산출하여 CFU/L로 제시하였다. 배양검사의 최소 검출한계는 200 CFU/L였다. 배양에서 확인된 의심 집락에 대한 균종 동정(16S ribosomal ribonucleic acid [16S rRNA] 및 macrophage infectivity potentiator [*mip*] 유전자 중합효소연쇄반응과 염기서열분석)은 전체 양성 집락에 일률적으로 적용하지 않고, 순환여과식 욕조수 검체와 역학조사관이 동정이 필요하다고 판단한 검체에 한하여 선택적으로 시행하였다.

### 3. 검출 정의 및 변수

검출(양성)은 선택배지 배양에서 레지오넬라균 집락이 확인된 경우로 정의하였고, 정량이 가능한 수계검체는 200 CFU/L 이상을 검출로 산출하였다. 검체 유형은 시험일지의 세부내역을 기준으로 냉각탑수, 온수, 욕조수, 냉수, 기타로 분류하였으며, 급수전과 샤워기 등 표면 도말검체는 기타에 포함하였다. 도말검체는 채수량이 없어 정량 환산이 불가하므로 정성 결과로 처리하였고, 이에 해당하는 양성 12건은 농도 분석에서 제외하였다. 농도는 정량값이 확인된 양성 검체 558건을 대상으로 1,000 CFU/L 미만, 1,000 CFU/L 이상–10,000 CFU/L 이하, 10,000 CFU/L 초과–100,000 CFU/L 미만, 100,000 CFU/L 이상으로 구분하였다. 구간 경계는 지침의 조치기준을 따라 정하였다. 2026년 지침은 냉각탑수와 급수시설 냉수와 온수에 서로 다른 단계별 조치를 제시하고, 목욕장 순환욕조수에는 1,000 CFU/L 수질기준이 적용되므로 농도구간은 검체 유형에 따라 해석하였다[6].

### 4. 통계 분석

검출률은 검출 검체 수를 검사 검체 수로 나누어 산출하였다. 검체 유형 간 차이는 Pearson 카이제곱검정으로 평가했고, 검체원 및 냉각탑수 검체원 비교의 2×2 표에는 Yates 연속성 보정을 적용하였다. 냉수를 기준으로 유형별 검출률비를 산출하였다. 전체 양성 중 냉각탑수가 차지하는 비중의 연도 추이는 Cochran–Armitage 경향검정으로 평가하였으며, 주요 검출률에는 Wilson 95% 신뢰구간을 제시하였다. 의뢰경로별 검정은 두 접수군의 검출률 분포를 비교하기 위해 시행했으며, 대상 선정과 채취 주체, 재검 방식이 다르므로 경로 자체의 효과나 채취 방식의 우열을 평가한 것으로 해석하지 않았다. 전체 양성 중 냉각탑수 비중은 연도별 검체 유형 구성의 영향을 받으므로 3년 합산 검체 유형 구성을 표준으로 한 직접표준화를 민감도분석으로 시행하고 관찰값과 함께 제시하였다. 직접표준화 비중은 각 연도의 검체 유형별 검출률을 3년 합산 검체 유형 구성에 적용하여 산출한 것으로 검체 구성이 매년 동일했다고 가정할 때 기대되는 냉각탑수 비중을 의미한다. 이를 통해 관찰된 비중 변화를 검체 유형 구성의 변화에 기인한 부분과 유형별 검출률의 변화에 기인한 부분으로 구분하고자 하였다. 직접표준화 비중의 95% 신뢰구간은 각 검체 유형 및 연도 층의 이항 재표집에 기반한 부트스트랩(bootstrap)으로 산출하였다. 분석은 Python 3.12 (Python Software Foundation)를 이용하였고 양측 p<0.05를 통계적 유의수준으로 하였다.

## 결 과

### 1. 전체 및 연도별 검출 현황

분석 대상 9,172건(관원 검체 3,643건, 민원 검체 5,529건) 중 570건(6.21%, 95% 신뢰구간 5.74–6.73%)에서 레지오넬라균이 검출되었다. 연도별 검출률은 2023년 6.98% (193/2,764건), 2024년 4.22% (137/3,246건), 2025년 7.59% (240/3,162건)로 경향성은 보이지 않았다. 관원 검체와 민원 검체의 3년 합산 검출률은 각각 6.73%와 5.88%였고 통계적으로 유의한 차이는 없었다(표 1). 검사목적별로는 관원 역학조사 검체가 9.82% (120/1,222건)로 가장 높았고, 민원 검체 5.88% (325/5,529건), 관원 예방검체 5.16% (125/2,421건) 순이었다. 연도별 값은 검체 유형과 의뢰 경로의 구성 변화가 반영된 보정하지 않은 비율이므로 기술적으로 제시하였다.

### 2. 검체 유형별 검출 현황

검체 유형별 검출률은 욕조수 18.30% (86/470건, 95% 신뢰구간 15.06–22.05%), 온수 11.03% (166/1,505건, 95% 신뢰구간 9.54–12.72%), 냉각탑수 5.53% (269/4,865건, 95% 신뢰구간 4.92–6.21%), 기타 3.53% (30/851건), 냉수 1.28% (19/1,481건)로 유형 간 유의한 차이가 있었다(p<0.001). 냉수 대비 검출률비는 욕조수 14.3배, 온수 8.6배, 냉각탑수 4.3배였다. 욕조수, 온수, 냉각탑수, 냉수의 순서는 3년간 같았으나, 기타의 순위는 연도별로 달랐다(표 2, 그림 1). 기타는 분수대수와 저수조수, 여과기수 등 성격이 다른 수계와 정량이 불가한 표면 도말검체가 함께 포함된 혼합 범주이다. 냉각탑수는 전체 검사량의 53.0%와 전체 양성의 47.2%를 차지하였다. 전체 양성 중 냉각탑수 비중은 2023년 34.2%, 2024년 43.8%, 2025년 59.6%로 유의하게 증가하였다(경향검정 p<0.001) (그림 2). 다만 냉각탑수가 전체 검사에서 차지하는 비중도 2023년 42.8%, 2024년 56.7%, 2025년 58.3%로 증가하였고 온수와 냉수의 검사 건수는 감소하였다. 3년 합산 검체 유형 구성으로 직접표준화한 냉각탑수 비중은 2023년 43.2%, 2024년 41.5%, 2025년 53.1%로 지속적으로 증가하지는 않았으나 2025년이 가장 높았다. 관찰된 증가분의 약 3분의 2는 검체 구성 변화에, 약 3분의 1은 유형별 검출률 변화에 기인하였다. 냉각탑수 검체 내 검출률의 연도별 추이는 유의하였다(2023년 5.58%, 2024년 3.26%, 2025년 7.75%; 경향검정 p=0.001).

### 3. 냉각탑수 검사 구성

냉각탑수 4,865건 중 4,691건(96.4%)은 민원 검체였고 174건(3.6%)은 관원 검체였다. 냉각탑수 양성 269건 중 243건(90.3%)은 민원 검체였다. 관원 냉각탑수 검출률은 14.94% (26/174건, 95% 신뢰구간 10.41–20.99%), 민원 검체는 5.18% (243/4,691건, 95% 신뢰구간 4.58–5.85%)였고 차이는 유의하였다(그림 3).

### 4. 균종 및 정량 농도

양성 570건 중 염기서열분석으로 균종이 동정된 131건에서는 *Legionella pneumophila* 112건(85.5%), *Legionella qingyii* 6건(4.6%), *Legionella spiritensis* 4건(3.1%), *Legionella anisa* 및 *Legionella pneumophila* subsp. *pascullei* 각 3건(2.3%) 등이 확인되었다. *L. pneumophila* subsp. *pascullei*를 포함한 *L. pneumophila* 계열은 115건(87.8%)이었다. 정량 환산이 가능한 양성 558건(도말검체 12건 제외)의 중앙값은 1,800 CFU/L (범위 200–1,024,000 CFU/L)이었다. 350건(62.7%)은 1,000 CFU/L 이상이었고, 10,000 CFU/L 초과 133건 중 냉각탑수는 74건(55.6%)이었다(표 3).

## 논 의

욕조수와 온수는 검출률이 가장 높았지만, 냉각탑수는 전체 검사량의 53.0%와 양성의 47.2%를 차지했고 전체 양성 중 비중도 2023년 34.2%에서 2025년 59.6%로 증가하였다(검체 구성 표준화 시 43.2%에서 53.1%). 이 연구가 제시하는 핵심은 검체당 검출률과 전체 양성 부담이 서로 다른 관리지표라는 점이다. 따라서 환경수계 관리의 우선순위를 단일 검출률 순위로 정하기보다, 검체당 검출 가능성과 양성 건수의 규모와 추이를 함께 평가해야 한다.

욕조수, 온수, 냉각탑수, 냉수의 검출률 순서는 3년간 일관되었고, 이는 따뜻하고 정체된 수계와 생물막이 레지오넬라균 증식에 적합하다는 기존 지식과 부합한다[3,4]. 욕조수와 온수는 검사량이 상대적으로 적더라도 검출 가능성이 높으므로 수온과 소독, 순환 및 청소를 중심으로 한 예방적 수계관리의 우선 대상이다. 다만 이 값은 접수 검체의 검출률이며 시설 수준의 오염률이나 감염위험을 직접 의미하지 않는다. 기타는 성격이 다른 수계와 정량이 불가한 표면 도말검체가 섞인 혼합 범주이므로 연도별 순위 변동을 단일 수계의 검출 특성으로 해석하지 않았다.

냉각탑수는 검출률 자체는 5.53%였지만 양성 건수와 고농도 양성에서 차지하는 비중이 컸고, 전체 양성 중 냉각탑수 비중이 3년간 유의하게 증가하였다. 이는 냉각탑수가 검출률 순위만으로는 드러나지 않는 감시 부담을 형성하고 있음을 보여준다. 또한 냉각탑수 검사의 96.4%와 양성의 90.3%가 자율의뢰 민원 검체였으므로 현재 확인되는 냉각탑수 양성 부담은 민원 수요에 크게 의존한다. 관원 냉각탑수에서 높은 검출률이 관찰된 점은 공무원의 대상 선정에 고위험 시설이 포함되었을 가능성을 시사한다. 다만 관원 냉각탑수는 174건으로 검사 건수가 적어 검출률의 95% 신뢰구간이 10.41–20.99%로 넓었으므로, 두 경로 간 대상 선정 및 채취 주체의 차이를 고려하면 이 차이를 정량적으로 확정하기는 어렵다. 관원 계획검사는 검사 건수의 일률적 확대보다는 시설 유형과 에어로졸 노출 가능성, 과거 검출 이력 등 위험요인에 따라 민원 자율의뢰로 포착되지 않는 고위험 시설을 표적 선정하는 방향을 고려할 수 있다.

정량 환산이 가능한 양성 558건 중 350건(62.7%)이 1,000 CFU/L 이상이었고, 10,000 CFU/L 초과 133건 중 냉각탑수가 74건(55.6%)을 차지하였다. 이는 냉각탑수의 중요성이 검사량뿐 아니라 고농도 양성 부담에서도 확인됨을 보여준다. 다만 냉각탑수, 급수시설 냉수와 온수, 목욕장 욕조수에는 서로 다른 조치기준이 적용되므로[6], 농도는 수계 유형별 기준에 따라 해석해야 한다. 동정 결과가 기록된 131건에서는 *L. pneumophila* 계열이 87.8%를 차지하였다. 다만 균종 동정은 순환여과식 욕조수와 역학조사 검체 등 일부에만 선택적으로 시행되었으므로, 확인된 균종 분포는 전체 양성 검체를 대표하지 않는다.

이 분석에는 다음과 같은 네 가지 제한점이 있다. 첫째, 단일 기관에 접수된 관원 검체와 자율의뢰 민원 검체를 분석했으므로 경기도 전체 시설의 오염률을 추정할 수 없다. 둘째, 관원 검체와 민원 검체는 대상 선정과 채취 주체, 재검 방식이 달라 의뢰경로별 검출률 차이를 인과적으로 해석할 수 없다. 셋째, 수온과 잔류염소, 배관 상태, 청소 이력 등 시설 관리변수를 분석하지 못해 검출 차이의 구체적 원인을 평가하지 못했다. 넷째, 전체 양성 중 냉각탑수 비중은 연도별 검체 구성의 영향을 받아 관찰된 증가의 상당 부분이 구성 변화로 설명된다(2023–2024년 상승은 표준화 후 소실되나, 2025년 비중은 표준화 후에도 가장 높았다). 따라서 이 비중은 연구원이 처리한 양성 부담의 구성을 기술하는 값이며, 냉각탑 시설의 오염 위험 증가를 직접 의미하지는 않는다.

결론적으로, 이 연구는 ‘어디에서 검출률이 높은가’와 ‘어디에서 양성 부담이 크게 축적되는가’가 동일하지 않음을 보여주었다. 욕조수와 온수는 예방적 수계관리의 우선 대상이고, 냉각탑수는 전체 양성의 가장 큰 비중을 차지하며 그 비중도 증가하고 있어 감시의 우선 대상이다. 따라서 환경수계 관리는 검체 유형별 검출률과 양성 건수의 규모와 추이를 함께 적용하는 이중 우선순위로 설계해야 하며 관원 계획검사는 민원 자율의뢰로 포착되지 않는 고위험 냉각탑을 보완하는 역할을 강화할 필요가 있다. 이러한 환경감시가 고위험 수계의 조기 확인과 선제적 조치로 이어질 때 레지오넬라증 환자 발생을 줄이는 데 기여할 수 있다.

## Figures and Tables

**그림 1:** 경기도보건환경연구원 접수 검체의 검체 유형별 레지오넬라균 검출률, 2023–2025년 합산 오차막대는 Wilson 95% 신뢰구간을 의미.

**그림 2:** 경기도보건환경연구원 접수 양성 검체 중 냉각탑수 비중의 연도별 관찰값과 직접표준화값, 2023–2025년 진한 막대는 관찰값(전체 양성 중 냉각탑수 비중이며, 막대 위에 비중과 냉각탑수 양성/전체 양성 건수를 표시)을, 옅은 색 막대는 3년 합산 검체 유형 구성으로 직접 표준화한 값을 나타냄. 오차막대는 95% 신뢰구간으로, 관찰값은 Wilson 방법으로, 직접표준화 값은 이항 재 표집에 기반한부트스트랩(bootstrap)으로 산출.

**그림 3:** 경기도보건환경연구원 접수 검체의 검체원별 냉각탑수 검사량과 검출률, 2023–2025년 (A) 냉각탑수 검사량, (B) 냉각탑수 검출률. 관원 검체 174건은 예방 173건과 역학조사 1건으로 구성, 오차막대는 Wilson 95% 신뢰구간을 의미.

**표 1:** 경기도보건환경연구원 접수 검체의 연도 및 검체원별 레지오넬라균 검사 및 검출 현황, 2023–2025년

검체원	연도a)	검사 건수	검출 건수	검출률(%)b)
관원 검체	2023	1,441	109	7.56
	2024	1,243	54	4.34
	2025	959	82	8.55
민원 검체	2023	1,323	84	6.35
	2024	2,003	83	4.14
	2025	2,203	158	7.17
전체	2023	2,764	193	6.98
	2024	3,246	137	4.22
	2025	3,162	240	7.59
	2023–2025	9,172	570	6.21

^a)^접수일 기준으로 연도 배정, 접수일이 결측된 1건은 접수번호를 기준으로 배정. ^b)^검출률=검출 건수/검사 건수×100.

**표 2:** 경기도보건환경연구원 접수 검체의 연도 및 검체 유형별 레지오넬라균 검출률, 2023–2025년

검체 유형	2023a)	2024	2025	전체 검사	검출률(%)
욕조수	27/138 (19.57)	34/180 (18.89)	25/152 (16.45)	470	18.30
온수	79/588 (13.44)	32/501 (6.39)	55/416 (13.22)	1,505	11.03
냉각탑수	66/1,182 (5.58)	60/1,839 (3.26)	143/1,844 (7.75)	4,865	5.53
냉수	5/583 (0.86)	2/496 (0.40)	12/402 (2.99)	1,481	1.28
기타b)	16/273 (5.86)	9/230 (3.91)	5/348 (1.44)	851	3.53

^a)^각 연도 칸은 검출 건수/검사 건수(검출률)를 나타냄. ^b)^기타에는 분수대수, 저수조수, 여과기수 등과 급수전 및 샤워기 표면 도말검체 포함.

**표 3:** 경기도보건환경연구원 접수 양성 검체의 동정 균종 및 정량 농도 분포, 2023–2025년

분류	건(%)
동정 균종(n=131)a)	
*Legionella pneumophila*	112 (85.5)
*Legionella qingyii*	6 (4.6)
*Legionella spiritensis*	4 (3.1)
*Legionella anisa*	3 (2.3)
*Legionella pneumophila* subsp. *pascullei*	3 (2.3)
기타b)	3 (2.3)
정량 농도 구간(n=558) (CFU/L)c)	
1,000 미만	208 (37.3)
1,000 이상 10,000 이하d)	217 (38.9)
10,000 초과 100,000 미만	117 (21.0)
100,000 이상	16 (2.9)

농도 구간은 기술적 분포로 수계 유형별 조치기준과 구분하여 해석해야 하며, 값은 건수(구성비, %). 반올림으로 인해 백분율의 합이 100%가 되지 않을 수 있음. CFU=colony-forming unit (집락형성단위). ^a)^양성 검체 중 동정 결과가 기록된 131건을 기준. ^b)^기타는 *Legionella bozemanii*, *Legionella erythra*, *Legionella micdadei* 각 1건. ^c)^양성 570건 중 정량 환산이 불가한 표면 도말검체 12건을 제외한 558건을 기준. ^d)^농도 구간은 상호배타적.
